# The Correlation Between Static and Dynamic Facial Asymmetry in Unilateral Cleft Lip and Palate

**DOI:** 10.1177/10556656241298143

**Published:** 2024-11-14

**Authors:** Christopher Wright, Philip Benington, Xiangyang Ju, Toby Gillgrass, Craig Russell, Ashraf Ayoub

**Affiliations:** 1 Orthodontic Department, Glasgow Dental Hospital & School, School of Medicine, College of Medical, Veterinary and Life Sciences, 3526 University of Glasgow, Glasgow, UK; 2 Medical Devices Unit, Department of Clinical Physics and Bioengineering, National Health Service of Greater Glasgow and Clyde, Glasgow, UK; 3 National Cleft Surgical Service for Scotland, 59842 Royal Hospital for Sick Children, Glasgow, UK; 4 Oral and Maxillofacial Surgery, Scottish Craniofacial Research Group, Glasgow University Dental Hospital and School, School of Medicine, College of Medical, Veterinary and Life Sciences, 3526 University of Glasgow, Glasgow, UK

**Keywords:** craniofacial morphology, facial cleft, imaging

## Abstract

**Objective:**

Assess the relationship between static and dynamic facial asymmetry in unilateral cleft lip and palate during maximum smile.

**Design:**

Retrospective cross-sectional study.

**Setting:**

Multidisciplinary dentofacial planning clinic.

**Participants:**

Thirty-one surgically managed non-syndromic unilateral cleft lip and palate patients between the ages of 13 to 17 years.

**Materials and Methods:**

Dynamic three-dimensional (3D) facial images (four-dimensional [4D]) throughout the course of a maximum smile were captured using video stereophotogrammetry at a rate of 60 frames per second, which generated 180 3D images/expression. A generic facial mesh containing more than 7000 vertices was superimposed onto the 3D facial images to quantify and track facial asymmetry throughout the captured sequence. Partial ordinary Procrustes analysis was utilized to calculate an asymmetry score at the rest position, maximum smile, and at the point of maximum mathematical asymmetry. The relationships between the asymmetry at rest and the asymmetry at the point of maximum smile (static 3D), and the maximum mathematical asymmetry (dynamic 4D) were evaluated.

**Results:**

Asymmetry scores were higher at maximum smile than at rest. Maximum mathematical asymmetry was observed in most cases during the relaxation phase. Static asymmetry at rest and maximum smile was strongly correlated with the maximum mathematical asymmetry (*r* = 0.941, *P* < .001).

**Conclusions:**

Static 3D asymmetry at both rest and maximum smile are strongly correlated with dynamic 4D facial asymmetry. The use of 4D imaging, combined with generic mesh conformation and dense correspondence analysis, provides a valid objective measure of facial asymmetry.

## Introduction

One of the stigmas of the surgically managed unilateral cleft lip and palate (UCLP) patient is the increased facial asymmetry with facial expressions. In the general population the chin tends to be the area of most notable facial asymmetry, but for cleft patients the asymmetry is more concentrated in the nasolabial region.^1^ This can result in social deprivation at an early age, due to teasing or bullying at school, the feeling of isolation in social situations, and difficulty in forming meaningful relationships with peers.^2^ Individuals may present with low self-esteem and self-worth; potentially arising from their lack of popularity at school, discrimination based on their appearance, and lack of social support.^3^ Research into the opinion of the cleft team on surgical outcome has suggested that patients receiving a high quality of care could still be left with residual facial asymmetry, altered speech, and negative long-term effects on their interpersonal skills.^4^

The assessment of facial asymmetry can be achieved via subjective or objective evaluation of two-dimensional (2D), three-dimensional (3D), or four-dimensional (4D) images. The final decision regarding the need for lip revision surgery is often determined by the subjective clinical assessment of the surgeon and may vary depending on the surgeon's confidence, competence, and willingness to perform the procedure. The main limitation of a subjective clinical grading of facial asymmetry is the poor inter-rater reliability, which highlights the need for more objective assessment measures.^5,6^

Assessment of facial asymmetry using 2D photographs has been commonly used in research studies and international comparative evaluations. However, 2D assessment underestimates the dynamic facial dysmorphology of facial expressions.^7^ The assessment of facial asymmetry using 3D imaging is becoming more widely used. However, there is currently no internationally agreed methodology for assessment and significant heterogeneity exists in the outcome measures used.^8^ One of the main limitations of 3D imaging is its inability to capture dynamic motion, and facial asymmetry has only been assessed at the point of maximum smile. Assessing the dynamics of facial expressions is therefore essential.^9^

Many studies have relied on the identification of a set of facial landmarks and subsequent linear and angular measurements to characterize facial morphology. However, this does not fully utilize the 3D captured surface data of the entire face.^5,10–12^ A more robust approach involves the use of a generic mesh consisting of a mathematical facial mask that contains over 7000 quasi-landmarks (vertices). This is superimposed (conformed) onto the 3D image to portray the comprehensive morphology of the face for analysis. This overcomes the limitations of the individual landmark-based analysis and allows the tracking of the vertices throughout the sequence of captured 3D frames.^13^ In this way, the facial muscle movements can be quantified for each expression, making the dynamic (4D) assessment of facial asymmetry in cleft lip and palate more informative than with static (3D) images alone.

It has been shown that facial asymmetry is not a constant and tends to be greater during facial expressions.^9^ Therefore, the evaluation of the dynamics of facial expressions, and the asymmetry of the related muscle movements, could potentially have an impact on the diagnosis and management of cleft-related residual deformities.

The barriers to incorporating 4D imaging into the routine assessment of facial asymmetry include the cost of the hardware (stereo-video cameras) and specialized software, as well as the storage of the generated data, and the complexity of the analysis. While current access to 4D imaging remains limited it would be advantageous to know if there is a relationship between static and dynamic facial dysmorphology in cleft lip and palate. This is beyond the capability of static 3D imaging, which does not record the dynamic nature of facial muscle movement.

Therefore, the aim of this study was to assess the possible relationship between static 3D asymmetry (at both rest and maximum smile) and dynamic 4D asymmetry (represented by the image of maximum mathematical asymmetry) in surgically managed UCLP cases.

## Materials and Methods

### Ethical Approval

Ethical approval was obtained from the Research Ethics Committee (REC) (REC Reference—22/SS/0090), and NHS Research and Innovation (R&I) approval was obtained (R&I Reference—GN220D246). A signed consent was obtained to include patient's photograph in this article.

### Sample Size Calculation

The level of significance, type I error (*α*) was set at 0.05, and type II error (*β*) was set at 0.20, the study was powered at 80% as per convention (1−*β*). As no previous research has been done on the correlation there is consequently no accurate estimation of the expected correlation coefficient in the literature. Due to the lack of similar studies in the literature, we estimated a correlation coefficient of *r* ≥ 0.5 between static (3D) and dynamic (4D) facial asymmetry in UCLP. Therefore, a sample size of 29 participants would ensure our study was adequately powered at 0.80 (80%) for all correlation coefficients greater than or equal to *r* ≥ 0.5.

### Participants

The study was carried out on thirty-one (31) surgically managed nonsyndromic UCLP cases, as patients with syndromic UCLP may exhibit additional facial characteristics indicative of increased facial asymmetry that could influence and confound the results.^14^ The age range of 13 to 17 years old was selected to ensure a homogenous sample and to target patients postprimary repair but prior to any orthognathic surgery.

### Data Capture

4D images were captured using a Di4D facial performance imaging system (Dimensional Imaging, Ltd. Hillington Park, UK) based on passive stereophotogrammetry. Passive stereophotogrammetry involves the capture of an object or surface with multiple synchronized cameras that produce stereo images that are combined and constructed into a 3D surface image.

The 4D imaging system captures 4D facial muscle movement data at a rate of 60-frames per second, which generates 60 3D facial images per second during facial animation. Maximum smile was captured over a period of 3 to 5 s at a rate of 60-frames per second; generating minimum of 180 3D images per expression. Each expression was then repeated and captured 3 times to ensure that the best sequence of images was used for analysis, as selected by a single independent reviewer. Maximum facial expressions were used for this study as they are more reproducible and standardized when compared to spontaneous facial movements or expressions.^15,16^

### Data Processing

**Figure 1.**
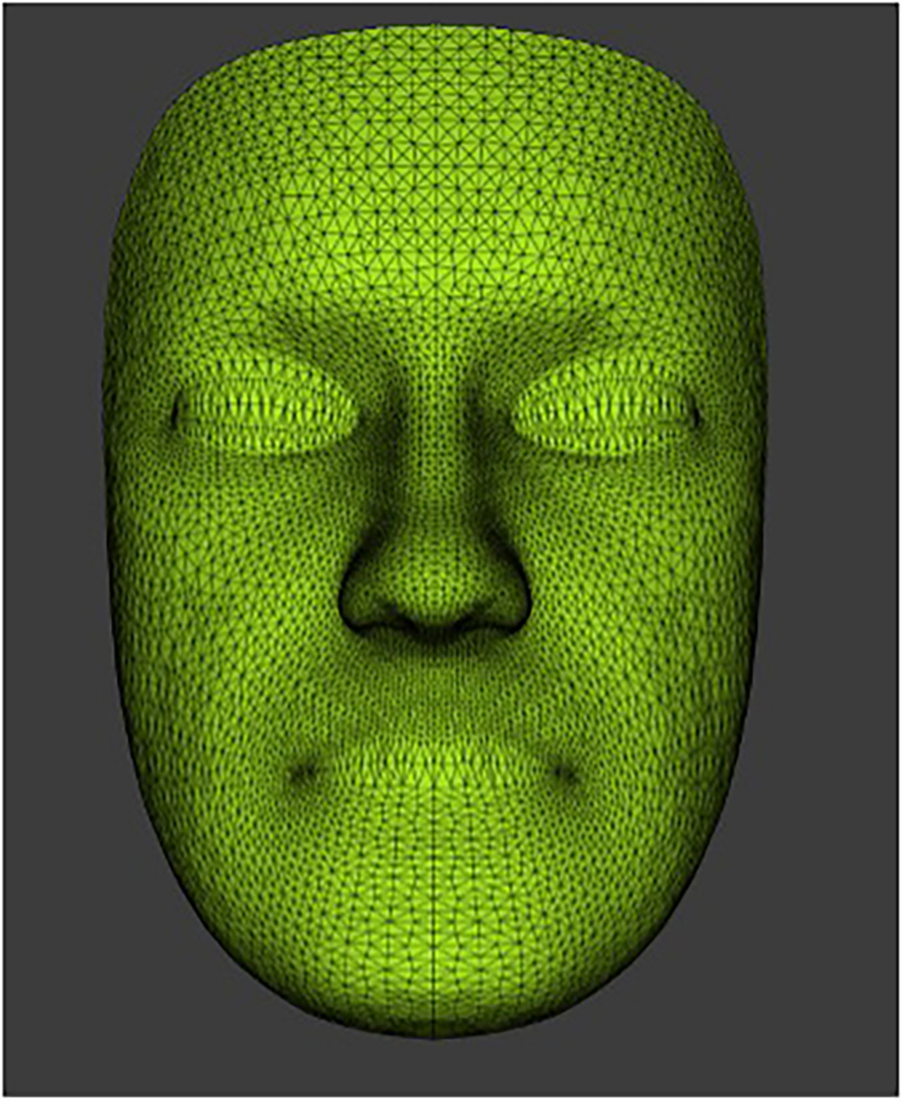
The generic mesh with over 7000 vertices or quasi-landmarks.

Each participant's data were processed in a standard sequence starting with the building of the raw data files. The next stage involved the conformation of the generic mesh (Figure 1). This is a mathematical mask containing over 7000 vertices, which are systematically spaced to cover the entire surface of the conformed mesh. Each vertex represents a fixed point with known coordinates in all planes of space (*x*, *y*, and *z*).

**Figure 2.**
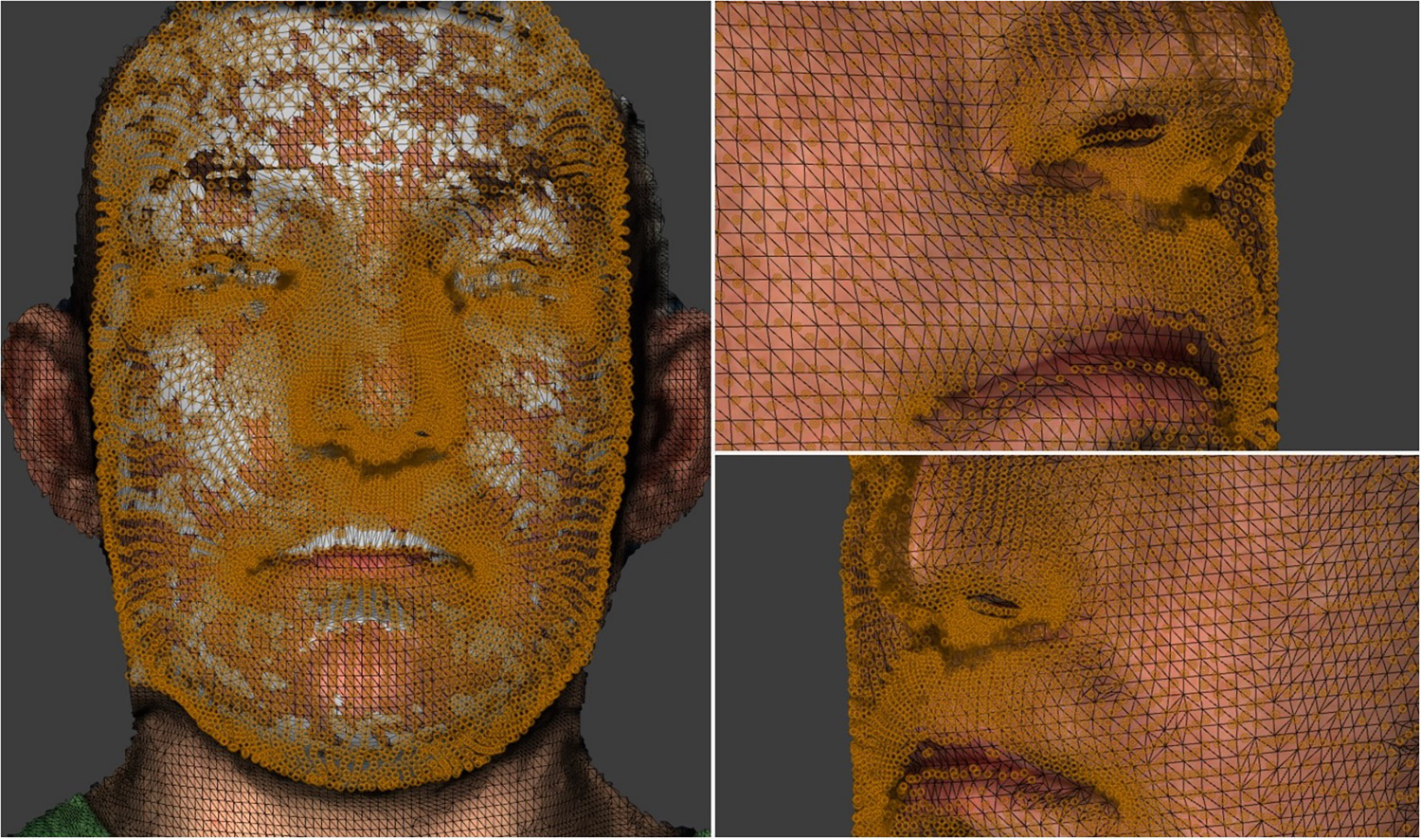
The density and distribution of the 7000+ vertices on the facial surface.

The generic mesh then undergoes an elastic deformation process (conformation) and is snapped onto the surface of each participant's facial image. The density and distribution of the 7000+ vertices are depicted in Figure 2. The mesh confirmation process initially requires the manual identification and labelling of 30 predetermined facial landmarks. The selected landmarks were distributed evenly around the generic mesh with increased landmark density around specific facial features of interest such as the mouth, eyes, and nose. Each landmark was fixed at a specific point on the generic mesh in 3D. Crucially, these landmarks were used solely in the generic mesh conformation process and were not used in the assessment of facial asymmetry in this study. Following the identification of the facial landmarks on the generic mesh the corresponding landmarks were also identified on the first frame of the facial expression of each participant. The Dimensional Imaging software was used to orientate and superimpose the generic mesh into the surface of 3D facial image of each case guided by the coordinated landmarks. This elastic deformation and transfer of the generic mesh then produces a unique conformed mesh for each participant which portrayed the 3D morphological characteristics of the face for analysis.

Previous research confirmed the accuracy of the mesh confirmation process. The mean absolute differences between the corresponding vertices mediolateral (*X*), vertical (*Y*), and anteroposterior (*Z*) directions of the repeated conformation of the generic mesh were 0.31, 0.27, and 0.29 mm, respectively.^9^

The conformed meshes were then automatically tracked throughout the course of a maximum smile for the assessment of facial asymmetry, and the 4D tracked mesh data were then saved and exported for data analysis. The process of automatic facial landmark tracking in 4D imaging has been previously validated.^17^ The 4D imaging technology allows automatic motion tracking of all 7000+ vertices on a subject's face. Data capture was performed advising subjects to minimize head motion, but any small head movements would have no effect on the analysis as each landmark is automatically tracked on the surface of the subject's face.

**Figure 3.**
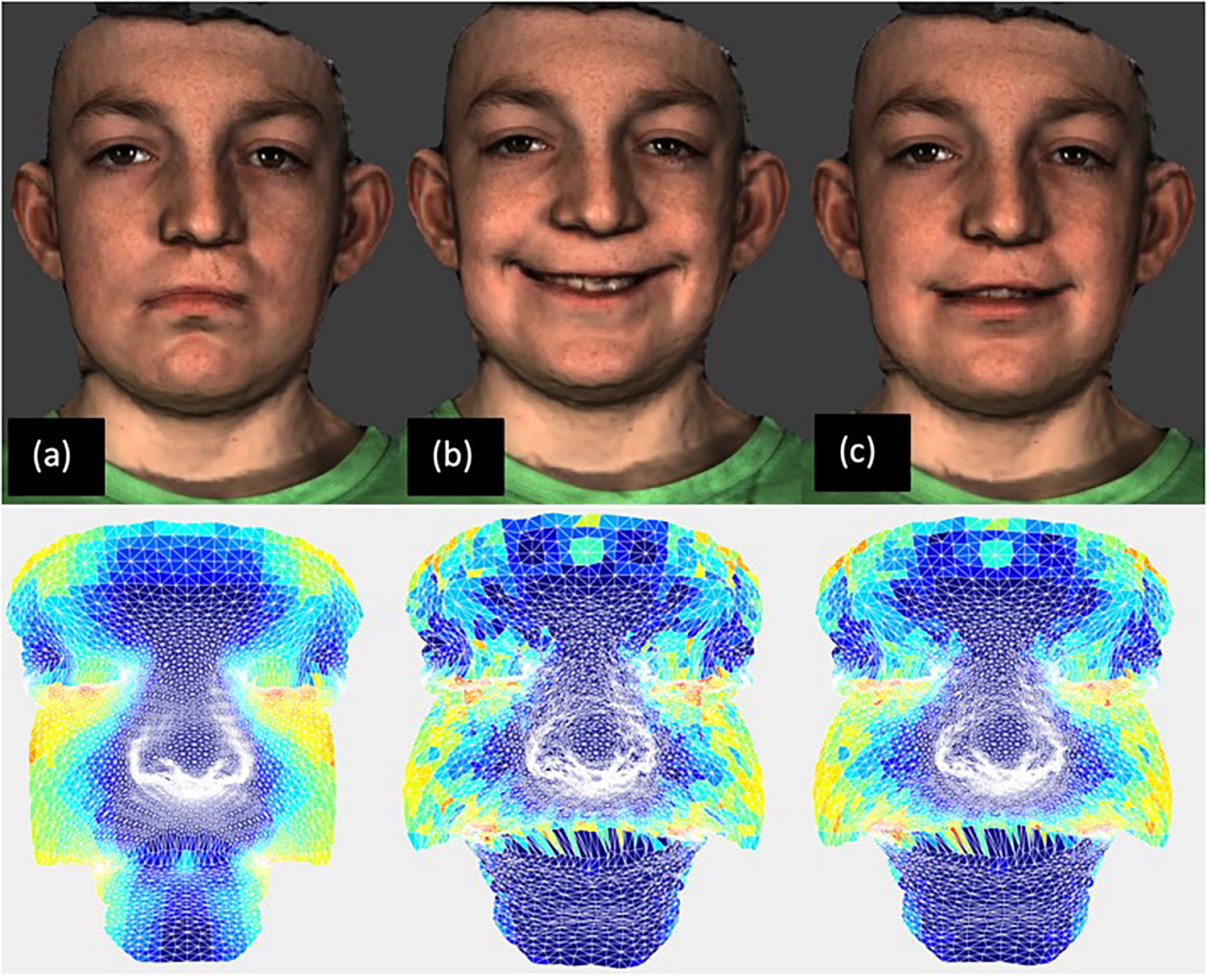
Frames used in analysis. Top row (a) rest frame (3D). (b) Frame of maximum smile (3D). (c) Frame of maximum mathematical asymmetry (4D). Bottom row shows the corresponding asymmetries, the color map illustrated the asymmetry scores from 0 to 5 mm as the colors from dark blue to dark red.

Abbreviations: 3D, three-dimensional; 4D, four-dimensional.

For each participant, every 3D image in the captured sequence was initially reflected in an arbitrary mathematical reference plane to create a mirror copy. The degree of facial asymmetry was then calculated using partial ordinary Procrustes analysis. The asymmetry score was based on the Procrustes distance, or the sum of the squared Euclidean distances between the conformed mesh and its own mirror image. Distance measurements were taken between all 7000+ vertices and their own corresponding mirror points. In perfect symmetry, the image and its mirror copy would superimpose exactly onto one another, with the sum of the squared distances between them being equal to zero. As facial asymmetry increases, the Procrustes distance and corresponding asymmetry score are also increased. Asymmetry scores were calculated for images at rest, at maximum smile, and at the point of maximum mathematical asymmetry. Detailed asymmetry analysis included the full face, nasolabial region, upper lip, and cheek. The full-face asymmetry score includes the sum of the squared Euclidean distances between all the 7000+ vertices, with regional analysis focusing on the quasi-landmarks located in the specific areas of interest only. The maximum asymmetry score was a measure of dynamic facial dysmorphology. The exact image with the maximum asymmetry score was also identified to determine at what point the greatest degree of dysmorphology occurred throughout the course of facial expression. Figure 3 shows the 3D images of a patient at rest, maximum smile, and maximum mathematical asymmetry.

### Statistical Methodology

The calculated asymmetry scores were assessed for normality using both graphical and numerical methods. As the data were normally distributed, Pearson's correlation coefficient (*r*) was used to explore the relationship between static 3D asymmetry (at both rest and maximum smile) and dynamic 4D asymmetry (represented by the image of maximum mathematical asymmetry).^18^

## Results

A total of 31 participants were included and the baseline characteristics of all participants are shown in Table 1. All participants were surgically managed non-syndromic UCLP cases. The mean age of participants was 14.61 which ranged from 13 to 17 years with a standard deviation of ±1.45.

**Table 1.** Patient Baseline Characteristics.

Baseline characteristic	Total (*n* = 31)
Age (mean ± SD, range)	14.61 ± 1.46, 13-17
Gender—Female (*n*, %)	11, 35.5%
Gender—Male (*n*, %)	20, 64.5%
Laterality—Left (*n*, %)	19, 61.3%
Laterality—Right (*n*, %)	12, 38.7%

The sample consisted of 20 males and 11 females. With regards to the laterality of the UCLP defect, left-sided clefting was more common making up 61.3% (*n* = 19) of the sample compared to only 38.7% (*n* = 12) cases of right-sided clefting.

Asymmetry scores were higher for the full-face, in comparison to the nasolabial region, the upper lip, and the cheek. This was independent of which image in the sequence was analyzed, that is, rest frame, frame of maximum smile, or frame of maximum mathematical asymmetry. The degree of asymmetry was greater at maximum smile in comparison to the image at rest. However, the asymmetry scores for the maximum smile image were less than those for the images of maximum mathematical asymmetry, as identified from the sequence of captured images.

Four patients exhibited maximum asymmetry during muscular contraction in the build-up to the maximum smile, while 27 patients exhibited maximum asymmetry during the relaxation phase.

For all regions of the face, the asymmetry scores for both the rest and maximum smile images were strongly positively correlated with the asymmetry scores of the images of maximum mathematical asymmetry (> 0.7). A summary of correlation coefficient results can be found in Table 2.

**Table 2.** Correlation Between Static 3D Asymmetry (at Both Rest and Maximum Smile) and Dynamic 4D Asymmetry (Represented by the Image of Maximum Mathematical Asymmetry).

			Asymmetry score rest (3D)	Asymmetry score maximum smile (3D)
Full face	Asymmetry score maximum mathematical asymmetry (4D)	Pearson correlation (*r*)	.898**	.911**
		Sig. (2-tailed)	<.001	<.001
Nasolabial region	Asymmetry score maximum mathematical asymmetry (4D)	Pearson correlation (*r*)	.922**	.941**
		Sig. (2-tailed)	<.001	<.001
Upper lip	Asymmetry score maximum mathematical asymmetry (4D)	Pearson correlation (*r*)	.766**	.796**
		Sig. (2-tailed)	<.001	<.001
Cheek	Asymmetry score maximum mathematical asymmetry (4D)	Pearson correlation (*r*)	.868**	.903**
		Sig. (2-tailed)	<.001	<.001

Abbreviations: 3D, three-dimensional; 4D, four-dimensional.

** Correlation is significant at the 0.01 level (2-tailed); *N* = 31.

**Figure 4.**
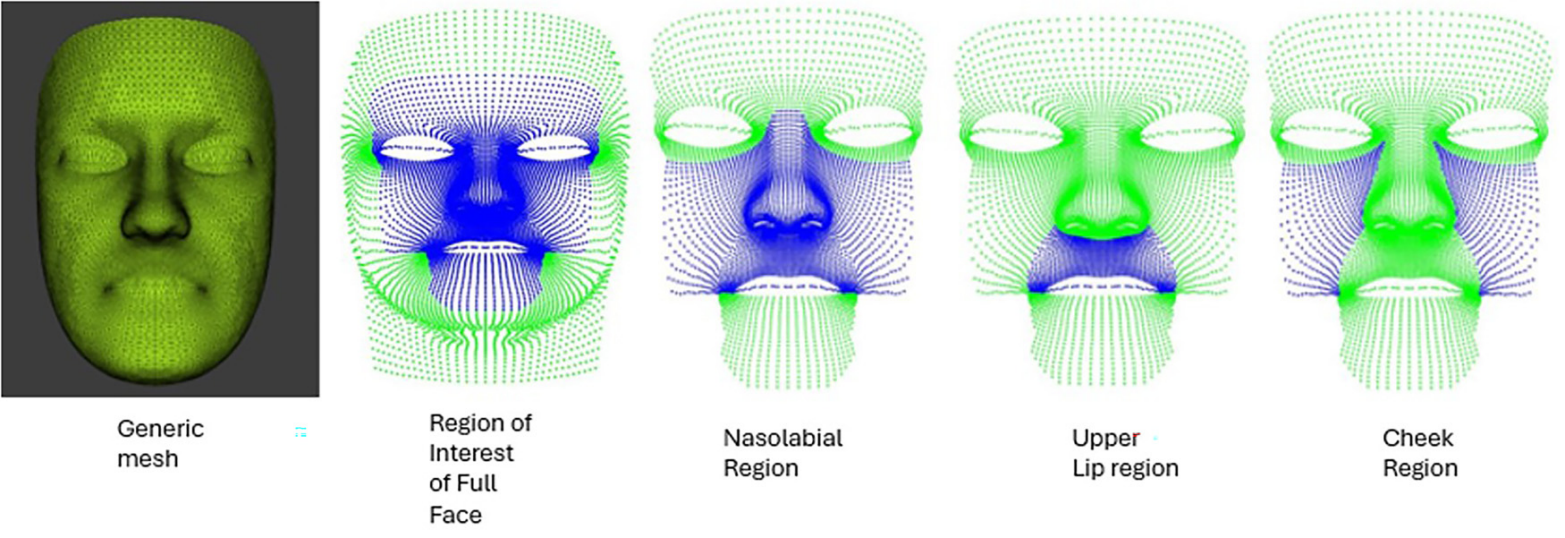
The anatomical regions of the face which includes the noso-labial, upper lip, and cheeks.

**Figure 5.**
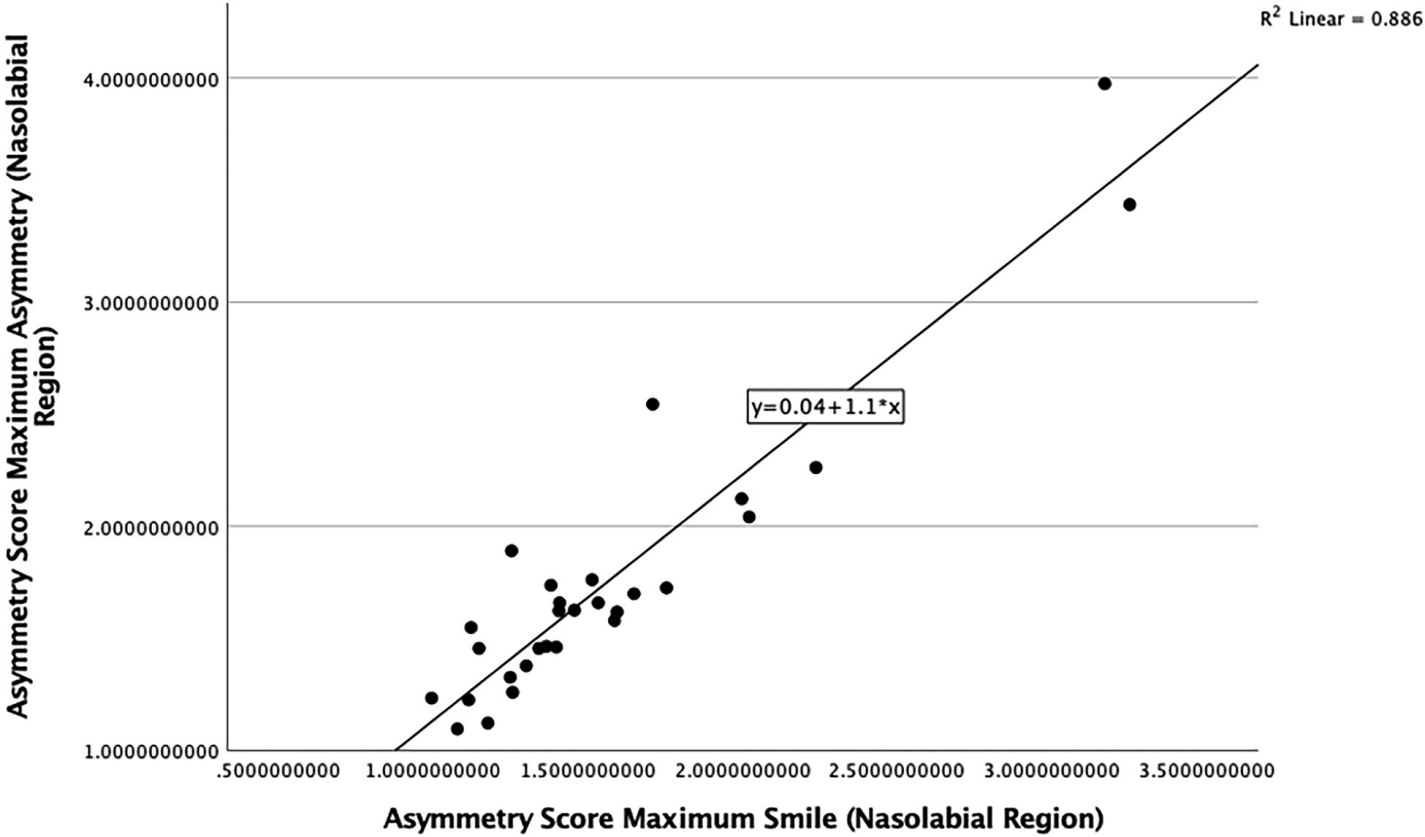
Scatterplot showing the correlation between asymmetry scores at maximum smile (3D) and maximum asymmetry scores (4D) (nasolabial region).

Abbreviations: 3D, three-dimensional; 4D, four-dimensional.

For all regions of the face (Figure 4), the maximum mathematical asymmetry score was more strongly correlated with the score at maximum smile than with the score at rest. For the nasolabial region, the strongest correlation was found between the asymmetry scores for the maximum smile and the maximum mathematical asymmetry (*r* = 0.941, *P* < .001) (Figure 5).

## Discussion

The rationale of this study was to provide a unique and novel assessment of the 4D facial asymmetry in a cohort of surgically managed UCLP cases to improve the diagnosis and management of residual dysmorphology. The need for objective assessment for facial asymmetry in cleft care has been well documented, and an appropriate tool may help shape future surgical decisions.^5^ A single 3D image of patients at maximum smile would provide an accurate measure of the residual dysmorphology, mainly asymmetry, of the nasolabial region of the surgically managed UCLP cases. Nevertheless, it does not describe the mechanism of the distorted muscle function of the maximum smile secondary to lip scarring. This was explained in detail in our previous studies.^13^

In our study, patients demonstrated the lowest overall asymmetry scores at rest. This is supportive of other research into the asymmetry of UCLP.^12,13^ Asymmetry scores were highest for full-facial analysis compared to regional analysis of the nasolabial region, upper lip, or cheek. This is independent of which 3D image is analyzed, including those recorded at rest, at maximal smile, and at the point of maximum mathematical asymmetry. This highlights the global facial asymmetry of the surgically managed UCLP cases beyond the nasolabial region.^19^

The degree of facial dysmorphology, as depicted by the asymmetry scores, increased during maximum smile compared to the rest image, which agrees with previous studies.^20,21^ In most of the cases in our study the greatest degree of facial asymmetry was identified during the relaxation phase after the peak of the expression (*N* = 27). This supports the findings of other studies, which confirm that the degree of asymmetry is increased during muscular relaxation compared to any other point of the maximum smile.^13^ The asymmetry scores for maximum smile were less than, but almost comparable to, the measured maximum asymmetry scores. This highlights that assessment of facial asymmetry at maximum smile is still a valid evaluation of facial dysmorphology, despite not depicting the full extent of the asymmetry. However, it is important to highlight that 4D imaging has the capability and sensitivity to identify the exact time point at which maximum asymmetry occurs. This highlights the role of the compensatory muscle effect in reducing residual dysmorphology of surgically managed UCLP cases. This informs the decision-making process regarding the need for lip revision and muscle repair, aimed at dealing with this asymmetry.

The strongest correlation between the asymmetry at rest and maximum mathematical asymmetry was seen in the nasolabial region at rest and at the peak of the smile. The nasolabial region is an area of particular interest in cleft lip and palate research and has been the focus of large national and international studies.^22–24^ The single strongest correlation in our study was in the nasolabial region between the maximum smile and maximum mathematical asymmetry images throughout the course of the expression (*r* = 0.941, *P* < .001). This would not have been possible to identify without the recording and analysis of the dynamics of facial expressions using 4D imaging technology. It is important to emphasize that in this study the main focus was the analysis of the asymmetry at rest and at maximum smile rather than the evaluation of the magnitude of the facial expression in the study by Hallac et al,^25^ 2020 who demonstrated, in 36 healthy pediatric volunteers, a significantly greater displacement in the oral commissure on the left side compared to the right (*P* < .05).

Due to the strong correlation between the asymmetry scores at various time points, it would be possible, in theory, to apply linear regression modelling to predict the degree of maximum smile asymmetry based on the static 3D images captured at rest and at maximum smile. However, this would not provide detailed information on the dynamic nature of dysmorphology, which may impact on the pattern, speed, and magnitude, of facial expressions. In addition, static 3D images could not reveal the interaction of various groups of muscles responsible for the measured asymmetry of the lip movements during maximum smile.

The highest coefficient of determination was found between maximum smile and the image of maximum mathematical asymmetry, for the nasolabial region (*r*^2^ = 0.89). This indicates that asymmetry of the nasolabial region at maximum smile accounted for 89% of the variance in dynamic facial asymmetry.

Facial asymmetry scores can be used pre and postoperatively to assess treatment outcomes, but if asymmetry is measured using static images taken at maximum smile, the true asymmetry is being underestimated.

This study was limited to the analysis of the global nondirectional asymmetry score. Assessing the directionality of the asymmetry along each of the Cartesian frames of reference (*x*, *y*, and *z*) may provide additional information regarding the cause of the residual asymmetry of surgically managed UCLP cases. This would also inform the decision-making process in relation to the need for additional corrective surgery.

## Conclusions

The use of 4D imaging combined with the application of the conformed mathematical mesh provides a comprehensive measure of facial asymmetry. Asymmetry at rest and at maximum smile is strongly correlated to, and likely predictive of, maximum mathematical asymmetry, which can only be detected using 4D imaging. This correlation was at its strongest in the nasolabial region at maximum smile. Future research on the application of linear regression modelling for the prediction of dynamic facial asymmetry should be considered.

## Supplemental Material

sj-pdf-1-cpc-10.1177_10556656241298143 - Supplemental material for The Correlation Between Static and Dynamic Facial Asymmetry in Unilateral Cleft Lip and Palate Supplemental material, sj-pdf-1-cpc-10.1177_10556656241298143 for The Correlation Between Static and Dynamic Facial Asymmetry in Unilateral Cleft Lip and Palate by Christopher Wright, Philip Benington, Xiangyang Ju and Toby Gillgrass, Craig Russell, Ashraf Ayoub in The Cleft Palate Craniofacial Journal

**Figure:** Video 1.
