# Supplementary material for: The Correlation Between Static and Dynamic Facial Asymmetry in Unilateral Cleft Lip and Palate
Source: Cleft Palate Craniofac J. 2024 Nov 14;63(2):210–7. doi: 10.1177/10556656241298143 (PMC12754019; doi:10.1177/10556656241298143)
Supplement: sj-pdf-1-cpc-10.1177_10556656241298143 - Supplemental material for The Correlation Between Static and Dynamic Facial Asymmetry in Unilateral Cleft Lip and Palate [file sj-pdf-1-cpc-10.1177_10556656241298143.pdf]

SAGE Publishing

Publication consent form – SAGE Publishing

This consent form is for the publication of individual-level information **("Personal Information")**—as described below—regarding a person, living or deceased.

Personal Information: Including demographic data; personal medical information such as description of symptoms and conditions, medical procedures/treatments and recovery; genetic pedigrees and other personal genetic information; photographs; images; videos; voice and other recordings; or any other personal representation.

Full name of individual whose information is to be published **(the "Subject")**:

Charlie Snoddon.

Usually, it is the Subject who would be expected to provide consent for the publication of their Personal Information. However, where the Subject is a minor, deceased or otherwise incapacitated, their parent/guardian, next of kin or legally authorized representative **(the "Proxy")** may provide consent for the publication of this information. Where consent for publication is required for more than one individual, separate consent forms should be used for each person.

By signing this form, I give my consent for Personal Information relating to the Subject to be published by SAGE Publishing, its licensees, and assigns.

Furthermore, I understand the following with respect to the use of the Subject's Personal Information:

1. Complete anonymity cannot be guaranteed, and the Subject may potentially be identified from the published Personal Information.
2. My consent for publication may be withdrawn at any point before publication, but once the Personal Information has been published (including online ahead of print) withdrawal will no longer be possible.
3. The Personal Information, in full or in part, may be published in print, freely online and in other formats, it may appear on non-SAGE websites, it may be translated and it may be used for commercial purposes.
4. Unless otherwise agreed in writing between the parties and detailed in this form, neither the Subject nor—where applicable—the Proxy, nor any other party will receive any financial benefit from the publication of the Personal Information or for the rights granted under this form.

Please tick the applicable box(es):

☐ I have seen and approved all of the work to be published that includes the Personal Information.

✔ I have not seen all of the work to be published that will include the Personal Information, but I agree to its publication nonetheless.

☐ If the work to be published contains any genetic pedigrees, I confirm that all relevant family members have been informed and have consented to the publication of this information.

Full name of signee: Patricia Snoddon. Charlie Snoddon.

Signed: [signature] . C.S.

Date: 8.5.24

Relationship to the Subject, if applicable (leave blank if the Subject is signing for her/him/themselves):

Mum

SAGE Publishing

For the person who obtained consent:

Please **DO NOT** submit the completed consent form to SAGE unless requested, owing to potential legal/privacy issues with sending and receiving confidential information. The original of the signed form should be held by the treating institution, and a copy of the form should be made available to SAGE if requested.

If Proxy consent has been obtained, please specify the reason:

NA

If the Subject or the Proxy will receive financial benefit from the publication of the Personal Information, please provide details:

NA

Full name of person who obtained consent:

TOBY JONATHAN GILLGRASS

Full name of institution:

GLASGOW DENTAL HOSPITAL + SCHOOL

Signed: [signature]

Date: 08 05 24
